# Corrigendum

**DOI:** 10.1111/iwj.13826

**Published:** 2022-10-28

In the article [1], acrylic terpolymer should be replaced with cyanoacrylate tetrapolymer throughout the article. The affected sentences should have been:

On page 862, under Abstract, second sentence: We conducted an open‐label cluster randomised trial to compare the effectiveness of a combined regimen of (1) specialised skin cleansers with disposable body wipes and (2) either a cyanoacrylate tetrapolymer (T1) or zinc oxide (T2) skin protectant against disposable body wipes and zinc oxide protectant (control) in promoting IAD healing and reducing the risk of deterioration.

On page 865, under section 2.5 Intervention, first sentence on fourth paragraph: Treatment 1 (T1) consisted of the skin cleanser (3M Cavilon No‐Rinse Skin Cleanser) and a cyanoacrylate tetrapolymer skin protectant (3M Cavilon Advanced Skin Protectant).

On page 871, under section 4.2 IAD deterioration, the first sentence of the second paragraph: Although only the intervention arms received skin cleansers, all three arms used the same body wipes to remove incontinent matter and received skin protectants, either using zinc oxide or cyanoacrylate tetrapolymer.

On page 871, under section 4.3, Implications for practice, the second sentence of the fourth paragraph: In addition, we found that either a zinc oxide or cyanoacrylate tetrapolymer skin protectant would be beneficial to prevent further skin deterioration.

On page 871, under section 4.3, Implications for practice, the fourth sentence of the fifth paragraph: While the cyanoacrylate tetrapolymer was only applied up to two times per patient, the greater ease of applying zinc oxide resulted in a negligible difference in the time taken.”

The authors confirm all results and conclusions of this article remain unchanged.
